# A bibliometric review of functional ingredients and their efficacy in developing functional biscuits

**DOI:** 10.12688/f1000research.148029.3

**Published:** 2024-12-16

**Authors:** Kshama Vishwakarma, Varalakshmi Chandra Sekaran, Vidya Patwardhan, Asha Kamath

**Affiliations:** 1 Welcomgroup Graduate School of Hotel Administration, Manipal Academy of Higher Education, Manipal, Karnataka, 576104, India; 2 Department of Health Policy, Prasanna School of Public Health, Manipal Academy of Higher Education, Manipal, Karnataka, 576104, India; 3 Department of Data Science, Prasanna School of Public Health, Manipal Academy of Higher Education, Manipal, Karnataka, 576104, India

**Keywords:** “Antioxidants” “Functional ingredients”, “Dietary fibers”, “Functional biscuits/cookies”, “Phenolic compounds”

## Abstract

**Introduction:**

Numerous studies have concluded that the functional ingredients benefit human health. Similarly, present times have seen exponential growth in functional food in bakery product segments like breads and biscuits. However, there is a lack of information on functional ingredients and their usefulness in developing functional bakery products. This bibliometric study addresses this gap by identifying the current research trends in functional ingredients.

**Objective:**

To investigate current research trends on functional ingredients and their usefulness in developing functional biscuits.

**Method:**

The study followed the “Scientific Procedures and Rationales for Systematic Literature Reviews” standards for retrieving literature. The study went through three major stages, “assembling,” “arranging,” and “assessing,” to retrieve 612 articles from the Scopus database from 2013 to 2023. Through further filtering, 395 articles were selected.

**Result:**

The analysis was conducted using R Studio and VOS viewer. The performance analysis and science mapping tools were used to evaluate the articles. The results showed a 5.76% annual growth in publication trends. The most researched functional ingredients were antioxidants, bioactive compounds, and dietary fiber. The review summarized the most studied foods to develop functional biscuits and highlighted the most experimented technological advancements.

**Conclusion:**

The study revealed the need for future research studies on functional ingredients with a focus on studying the technical implications of technical advancements in extracting functional ingredients from foods. The study highlights the significance of future studies based on the acceptance of functional biscuits and their sensory properties, focusing on the mass population. The study derives the possible applicability of functional ingredients in developing new formulations from publications and their usefulness in developing new formulations. This insight on the applicability of functional ingredients provides an opportunity for biscuit/cookie manufacturing to boost consumption among the population to a new ascending graph.

## 1. Introduction

The 75th anniversary of World Health Day, celebrated in 2023, emphasized the theme “Health for all”, as promoted by the World Health Organisation, thereby underscoring the significance of research on food and nutrition. The studies on functional food have highlighted the influence of consumers on determining food choices based on health benefits. ^1^ This societal shift has led to a heightened interest in functional foods with functional ingredients that provide additional physiological benefits beyond their basic nutritional content. A study ^2^ defines functional foods as “foods enriched with nutrients to add health benefits, not for balanced calorie content”. Similarly, the International Food Information Council (2006) and the International Life Sciences Institute (1999) have commonly accepted the definition of “functional foods” as “Foods or parts of foods that provide additional physiological benefits beyond their basic nutritional value”. Additionally, the Food and Nutrition Board (FNB) of the National Academy of Sciences defines functional foods as products that products that include potentially health-promoting ingredients, such as “any modified food or food ingredient that may provide a health benefit beyond that of the traditional nutrients it contains.” In summary, functional foods are foods that promote good health, enhance well-being, and improve quality of life. Bakery products, including breads and biscuits, have been the subject of numerous studies to enhance their functional properties. The widespread consumption of biscuits, particularly in regions with the highest per capita consumption, makes them ideal candidates for enrichment. Moreover, the convenience, cost-effectiveness, nutrient effectiveness, and variation in taste of biscuits make them the most consumer-preferred product. ^2^ Past studies ^3^^–^^8^ have examined the incorporation of functional ingredients in biscuit formulations. Despite this, biscuits remain a popular choice for experimenting with increasing protein and improving other nutritional aspects. ^6^ Recent research has shown that certain dietary components, while not essential for survival, can significantly affect overall quality of life. These positive health effects are attributed to the biological or physiological activity of ingredients in the body and are referred to as “bioactive function ingredients” or “functional food ingredients”. ^9^ Functional ingredients are added to foods to promote or enhance their positive impact on human health. These ingredients are labeled “functional ingredients” because they offer expected health benefits through physiological changes in the human body. ^2^ The role of functional foods in promoting human health and reducing the risk of illness has led to a growing acceptance and an era of new functional foods based on functional ingredients. ^10^ Dietary fiber and phenolic compounds are two significant components found in plants with several physiological effects. These compounds are structurally different, and substances like phenolic acids, flavonoids, lignans, and stilbene are classified as functional ingredients due to their benefits beyond just nutritional and energetic gains. ^2^

The literature review indicates that research on functional ingredients has become increasingly significant due to consumers’ growing awareness of healthy food consumption. Among functional foods, functional biscuits have garnered considerable attention from researchers due to their widespread popularity among consumers. This trend allows biscuit producers to create nutritious and novel biscuits with health-enhancing qualities. The health benefits associated with functional biscuits are supported by numerous studies that incorporate functional ingredients, such as dietary fibers, phenolic compounds, antioxidants, and bioactive compounds. ^5^^,^^7^^,^^11^^,^^12^ These studies have highlighted various health benefits, including reducing obesity, lowering the risk of developing coronary disease, and preventing chronic and nutrition-related diseases, including nutrient deficiencies, diabetes, obesity, cardiovascular diseases, and cancers, among others. The present study aims to analyze publications from 2013 to 2023 on functional ingredients and provide an overview of the literature related to functional ingredients. The study investigates research trends in functional ingredients to understand their applicability in developing new formulations of functional biscuits. To achieve this, the study uses bibliometric analysis ^13^ to examine research trends in functional ingredients. The study’s scope includes terminology related to functional ingredients, such as bioactive compounds and functional food ingredients, and studies examining the incorporation of functional ingredients in biscuits/cookies, breads, etc.

**Research questions are framed as below:**

RQ1. What trend has been observed in recent years regarding research on functional ingredients?

RQ2. Which functional ingredients influenced the articles on functional ingredients study the most?

RQ3. What information can be gleaned from documents about the most prolific authors, influential articles, and contributing journals in functional ingredient research?

RQ4. What foods have been investigated as the focus of the majority of studies on functional ingredients in the development of functional biscuits in the past three years?

RQ5. Which functional ingredients have been the subject of most research publications?

RQ6. What potential advancements and functional ingredients could future research studies explore, focusing on their usefulness in developing functional biscuits?

## 2. Methods

**Figure 1.**
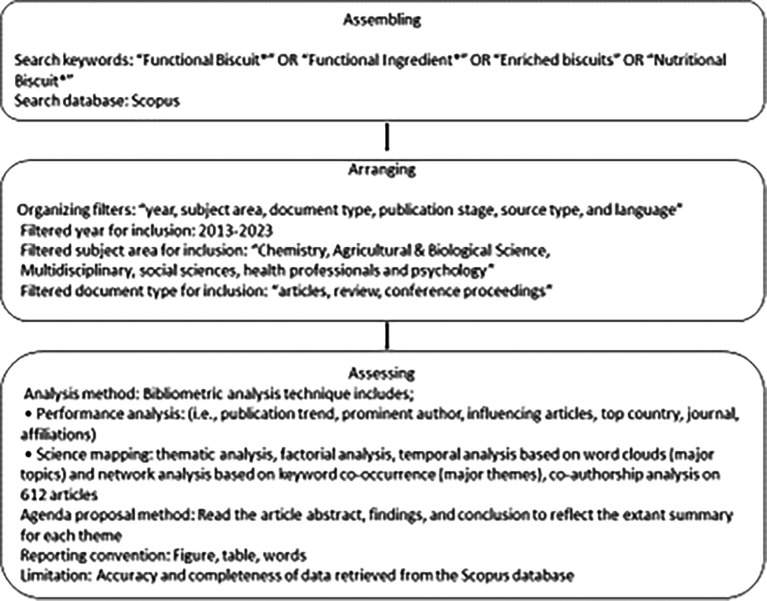
Scientific Procedures and Rationales for Systematic Literature Reviews Criteria.

The study predominantly adopts “Scientific Procedures and Rationales for Systematic Literature Reviews” (“SPAR-4-SLR”) standards ^14^ for retrieving literature, as illustrated in Figure 1.

The justification for choosing this method is the superior quality of SPAR-4-SLR ^14^ over the PRISMA Guidelines. Although PRISMA and PRISMA-P are both relatively comprehensive, enabling researchers to report their reviews in an orderly, rigorous, and transparent manner, they were developed for systematic reviews in general. They provided limited rationales researchers could use to justify their review decisions. ^14^ The SPAR-4-SLR protocol consists of three stages, including a. “Assembling,” involving identifying and obtaining literature that is not synthesized; b. “Arranging” involves organizing and refining literature under synthesis; c. “Assessing” involves assessing and presenting synthesized literature. ^14^ The current study was based on a Bibliometric analysis of the literature, and the rationale for following SPAR-4-SLR was to get logical and transparent reporting. In conclusion, while PRISMA is crafted for systematic reviews that focus on synthesizing evidence, SPAR-4-SLR is developed explicitly for reviews that aim to uncover research trends, identify gaps, and recognize patterns, often incorporating bibliometric analysis techniques.

### 2.1 SPAR-4SLR

***2.1.1 Assembling***

The study reviewed previous intellectual research work to get the required understanding to detect and obtain the range of articles on functional ingredients from the SCOPUS database. The alternate terminologies of functional biscuits, such as “enriched biscuits,” “functional cookies,” and “nutritional biscuits”, are included. The keywords “Functional biscuits” OR “Functional ingredients” generated 5187 documents in the first search approach. The second search approach followed the keywords “Functional biscuits” OR “functional ingredients” OR “Nutritional Biscuits,” which retrieved 5197 documents. The last approach used “Functional biscuits,” “Functional ingredients,” “Enriched Biscuits,” OR “Nutritional Biscuits,” generating 5265 documents. The document search was based on selected keywords, and the last approach was made on 17th February 2023 to collect articles. The search thread followed the title, abstract, and keywords to identify documents. The final search string result generated 5265 articles that were used for the further filtering process.

### 2.2 Arranging

Post the assembling step, the selected documents were arranged based on filter functions on the Scopus database as per “year, subject, document type, source type, publication stage, and language”. The search strategies were confined to “2013-2023. The subjects included “ *Chemistry, Agricultural & Biological Science, Multidisciplinary, Social sciences, Health professionals and Psychology*,” The type of documents included *Articles, Review, Conference proceedings, final, journal”*, and language for selected article was “ *English*,” respectively. The process yielded 612 articles. Furthermore, the data was downloaded in “CSV” format. Each article was read with a special emphasis on abstract, findings, and conclusion yielding 395 corpora of documents for review using databases such as Elsevier, Sage, Springer, Emerald, Taylor & Francis, Sci Vi, Wiley Open access, and Google Scholar. The document’s sources included the *1.“British Food Journal, 2. Food and Function, 3. Food and Nutrition Research, 4. Food Chemistry, 5. Food Research International, 6. Food Research, 7. Food Science and Nutrition, 8. Foods, 9. Foods and Raw Materials, 10. International Journal of Food Science and Technology, 11. Future Foods, 12. Journal of Food Processing and Preservation, 13.Journal of Food Science and Technology, 14. Journal of Functional Foods, 15. Nutrition and Food Science and Nutrients”.*

### 2.3 Assessing

The bibliometric analysis approach was employed to assess the collection of 395 articles on the functional ingredients research. Biblioshiny (Rstudio-R version 4.2.2) and VOSviewer (1.6.19) software were used as the assessment tool, using performance analysis and science mapping bibliometric analysis. ^15^ The analysis by Biblioshiny reveals year-wise publications, most influencing articles, most contributing journals, and prominent authors under performance analysis. VOS viewer displays the major keyword themes found in functional ingredients research and authors. ^13^^,^^15^

## 3. Results

### 3.1 Performance analysis

**Figure 2.**
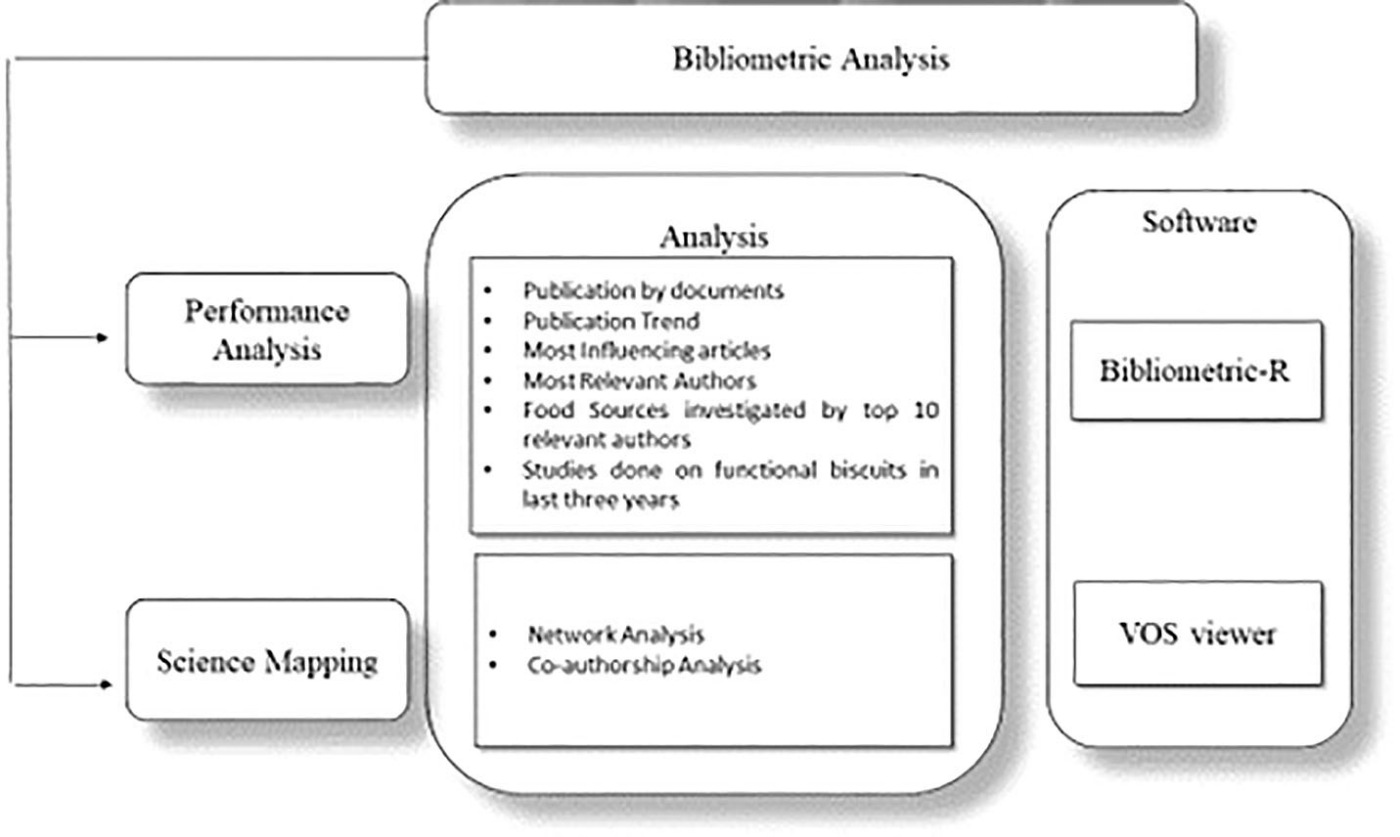
Bibliometric Tools Used in Analysis.

The performance of a specific research field is revealed through performance analysis. ^13^ Additionally, this analysis outlines publications through the years, trends of publications, most influencing articles, top contributing authors, and the most relevant authors. Figure 2 displays the study tools used for analysis.

### 3.2 Publication through the years

**Figure 3.**
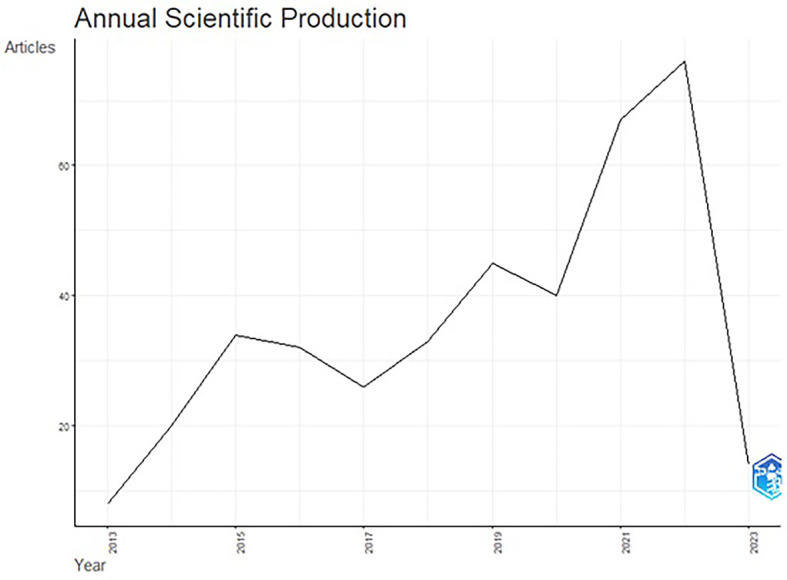
Publication Trends over the Years.

**Figure 4.**
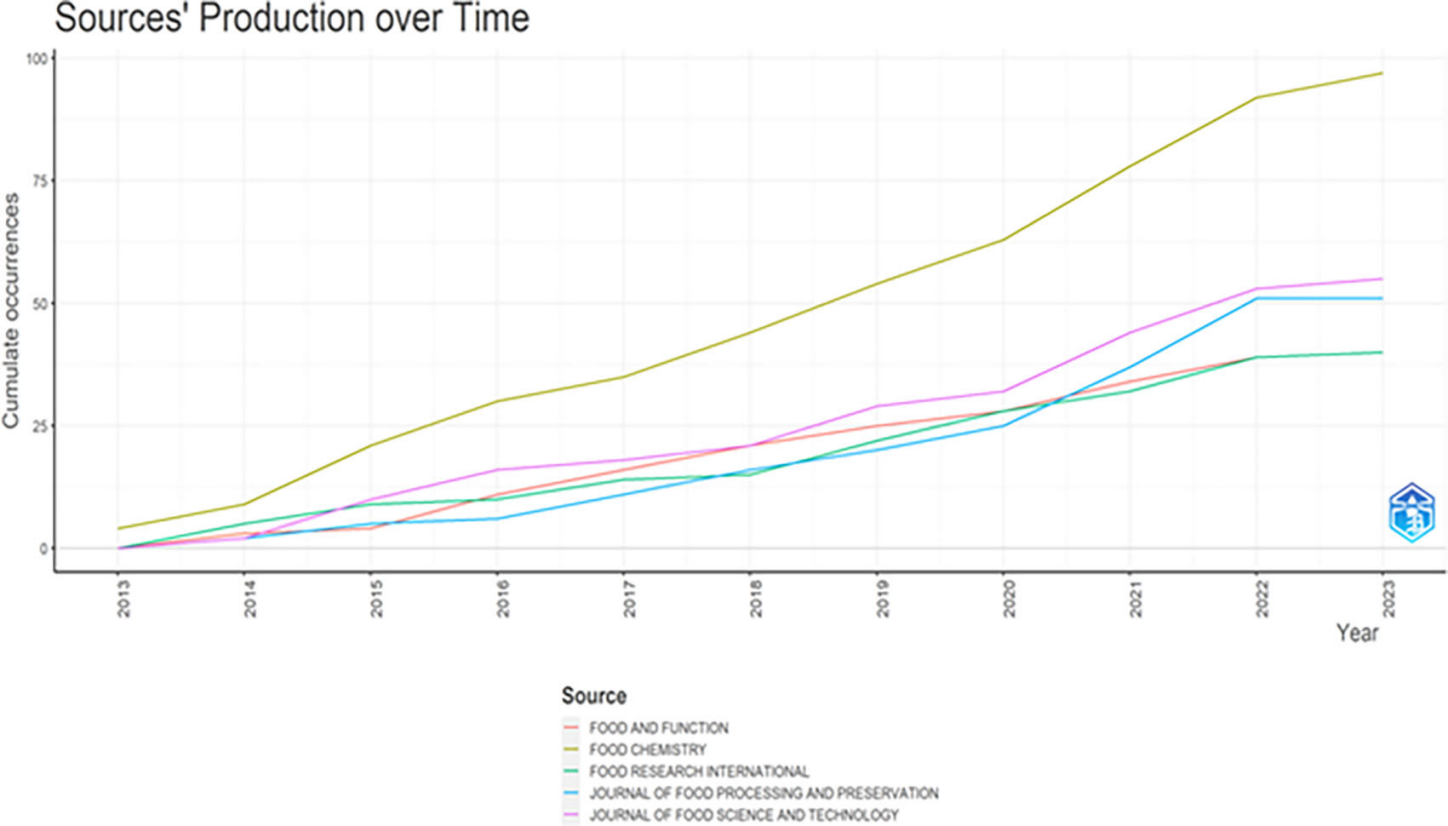
Sources Production over Time.

The total publication trend through 2013-2023 is represented in Figure 3, and Figure 4 illustrates publication trends over a decade. The publication trend from 2015 to 2022 is ascending in the number of articles published. The publication of articles increased close to double during the years 2021 & 2022. An upward graph has continued until 2022, although the annual growth rate remained fixed at 5.76%. In 2022, the number of published articles reached 76, and 2023 will likely see a further increase in research trends in functional ingredients. This rise in publications could be associated with COVID-19 and consistent efforts by the WHO to spread awareness of healthy food consumption and the development of alertness among the population about food choices.

The study examines diverse documents, including research on functional biscuits and ingredients. Table 1 represents publications with the keywords “functional ingredients,” “functional biscuits,” and “Nutritional biscuits.” The displayed statistics highlight 84.31% of documents in a category as published articles. Publications as review papers constitute 61% and 0.25% as conference papers.

**Table 1.** Publications by document types.

Document Type	Number of Publication	Percentage of Total Publication
Article	333	84.31%
Review	61	15.44%
Conference Paper	1	0.25%
Total	395	100%

### 3.3 Most influencing articles

Table 2 represents the most influential articles on functional ingredients research reaching a total number of citations above 75. It is observed that study ^16^ titled “ *HPLC-DAD-ESI-MS/MS screening of bioactive components from Rhus coriaria L. (Sumac) fruits.*” is the most prominent study on functional ingredients research published in the Food Chemistry with 331 total citations and 36.8 total citations per year. The article by Ref. 17 with a total of 288 citations, secured the second spot. Similarly, the top ten most cited articles have accumulated 2113 citations. The ten most influential articles ( Table 2) have studied sumac, peanuts, bananas, banana peels, brown algae, microalgae, mango peel, wheat, and oats as natural sources of bioactive compounds, dietary fiber, phenolic compounds, antioxidants, flavonoids, carotenoids, etc. Table 5 enlists food investigated by the most influential articles. The most cited articles set the foundation for future studies and innovation. Hence, future studies could contribute to optimization studies of functional ingredients (dietary fibers, bioactive compounds, phenolic compounds, etc.) to develop healthier and marketable functional biscuits.

**Table 2.** Most influential articles.

Author	Year	Source	Title	DOI	Total Citations	TC per Year
ABU-REIDAH IM	2015	Food Chemistry	*“HPLC-DAD-ESI-MS/MS screening of bioactive components from Rhus coriaria L. (Sumac) fruits.”*	10.1016/j.foodchem.2014.06.011	331	36.78
NEO YP	2013	Food Chemistry	*“Encapsulation of food grade antioxidant in natural biopolymer by electrospinning technique: A physicochemical study based on zein-gallic acid system.”*	10.1016/j.foodchem.2012.09.010	288	26.18
SINGH B	2016	Food Chemistry	*“Bioactive compounds in banana and their associated health benefits - A review”*	10.1016/j.foodchem.2016.03.033	229	28.63
ARYA SS.	2016	International journal of food science and technology	*“Peanuts as functional food: a review”*	10.1007/s13197-015-2007-9	217	27.13
KADAM SU.	2015	International journal of food science and technology	*“Extraction, structure and biofunctional activities of laminarin from brown algae.”*	10.1111/ijfs.12692	195	21.67
MATOS J	2017	Food and function	*“Microalgae as healthy ingredients for functional food: A review.”*	10.1039/c7fo00409e	190	27.14
QUIRÓS-SAUCEDA AE	2014	Food and function	*“Dietary fiber and phenolic compounds as functional ingredients: Interaction and possible effect after ingestion.”*	10.1039/c4fo00073k	182	18.2
VO T-S	2013	Journal of functional foods	*“Fucoidans as a natural bioactive ingredient for functional foods.”*	10.1016/j.jff.2012.08.007	166	15.09
ZHU F	2017	Food Chemistry	*“Encapsulation and delivery of food ingredients using starch based system.”*	10.1016/j.foodchem.2017.02.101	159	22.71
BUONO S	2014	Food and Function	*“Functional ingredients from microalgae.”*	10.1039/c4fo00125g	156	15.6
TORINO MI	2013	Food Chemistry	*“Antioxidant and antihypertensive properties of liquid and solid state fermented lentils.”*	10.1016/j.foodchem.2012.09.015	149	13.55
LUNA-VITAL DA	2015	Food research international	*“Biological potential of protein hydrolysates and peptides from common bean (Phaseolus vulgaris L.): A review”*	10.1016/j.foodres.2014.11.024	131	14.56
WANG L	2015	Food Chemistry	*“Preparation and physicochemical properties of soluble dietary fiber from orange peel assisted by steam explosion and dilute acid soaking.”*	10.1016/j.foodchem.2015.03.112	125	13.89
LIMÓN RI	2015	Food Chemistry	*“Fermentation enhances the content of bioactive compounds in kidney bean extracts.”*	10.1016/j.foodchem.2014.09.084	122	13.56
MŁYNARCZYK K	2018	Journal of functional foods	*“Bioactive properties of Sambucus nigra L. As a functional ingredient for food and pharmaceutical industry.”*	10.1016/j.jff.2017.11.025	112	18.67
BRESCIANI L	2014	Food research international	*“Phenolic composition, caffeine content and antioxidant capacity of coffee silverskin.”*	10.1016/j.foodres.2013.10.047	98	9.8
PASQUALONE A	2014	Food research international	*“Physico-chemical, sensory and volatile profiles of biscuits enriched with grape marc extract.”*	10.1016/j.foodres.2014.07.014	95	9.5
HUA M	2019	Food Chemistry	*“Analysis and determination of phytosterols and triterpenes in different inbred lines of Djulis (Chenopodium formosanum Koidz.) hull: A potential source of novel bioactive." ingredients*	10.1016/j.foodchem.2019.01.114	94	18.8
SȨCZYK Ł	2016	Food Chemistry	*“Effect of carob (Ceratonia siliqua L.) flour on the antioxidant potential, nutritional quality, and sensory characteristics of fortified durum wheat pasta.”*	10.1016/j.foodchem.2015.08.086	94	11.75
PASQUALONE A	2015	Food Chemistry	*“Production and characterization of functional biscuits obtained from purple wheat.”*	10.1016/j.foodchem.2015.02.025	94	10.44
NUNES JC	2016	Food Chemistry	*“Effect of drying method on volatile compounds, phenolic profile and antioxidant capacity of guava powders.”*	10.1016/j.foodchem.2015.11.050	91	11.38
PASTORIZA S	2017	Food and Function	*“Healthy properties of green and white teas: An update”*	10.1039/c7fo00611j	91	13
GAO Y	2018	International Journal of food science and Technology	*“Gluten-free bakery and pasta products: prevalence and quality improvement.”*	10.1111/ijfs.13505	91	15.17
NOOSHKAM M	2020	Food research international	*“Functional and biological properties of Maillard conjugates and their potential application in medical and food: A review”*	10.1016/j.foodres.2020.109003	90	22.5
LUO Y	2014	Food Chemistry	*“Physical, chemical and biochemical properties of casein hydrolyzed by three proteases: Partial characterizations.”*	10.1016/j.foodchem.2014.01.048	90	9
YU G	2018	Food Chemistry	*“Modification of carrot (Daucus carota Linn. var. Sativa Hoffm.) pomace insoluble dietary fiber with complex enzyme method, ultrafine comminution, and high hydrostatic pressure.”*	10.1016/j.foodchem.2018.03.037	87	14.5
MOCAN A	2016	Journal of functional foods	*“Biological and chemical insights of Morina persica L.: A source of bioactive compounds with multifunctional properties.”*	10.1016/j.jff.2016.05.007	85	10.63
DAOU C	2014	International Journal of food science and Technology	*“Functional and physiological properties of total, soluble, and insoluble dietary fibres derived from defatted rice bran.”*	10.1007/s13197-013-0925-y	78	7.8
GÓMEZ-MASCARAQUE LG	2017	Food Chemistry	*“Microencapsulation structures based on protein-coated liposomes obtained through electrospraying for the stabilization and improved bioaccessibility of curcumin.”*	10.1016/j.foodchem.2017.04.133	77	11
SINGH JP	2016	International Journal of food science and technology	*“Development of eggless gluten-free rice muffins utilizing black carrot dietary fibre concentrate and xanthan gum.”*	10.1007/s13197-015-2103-x	77	9.63

### 3.4 Most contributing journals

Table 3 displays the most contributing journals in functional ingredient research. It is evident that” Food Chemistry” and “Food & Function” journals stand first and second with “97” and “40” significant contributions, respectively. Interestingly both journals have amassed total citations “4144” and “1329” and stand at “39” and “19” *h index* individually with their higher contributions.

**Table 3.** Top most contributing journals.

Sources	Articles	Total Citations	h-index
Food Chemistry	97	4144	39
Food and Function	40	1329	19
Food Research International	40	1137	19
Journal of Food Science and Technology	55	982	17
International Journal of Food Science and Technology	34	706	13
Foods	26	284	10
Journal of Food Processing and Preservation	51	305	10
Nutrients	16	265	10
Journal of Functional Foods	10	554	8

### 3.5 Most prolific authors

**Figure 5.**
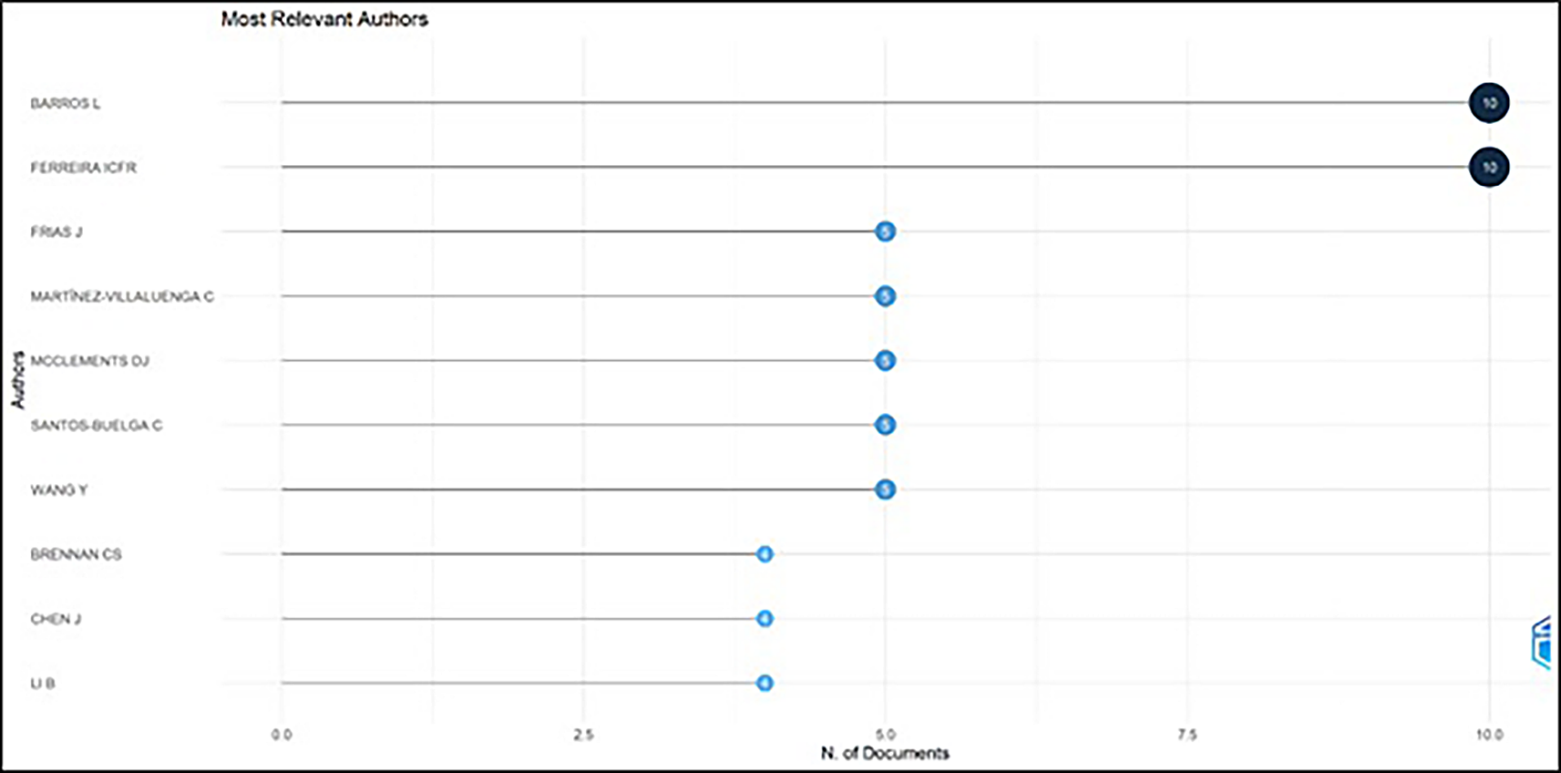
The Most Prolific Authors.

Table 4 and Figure 5 exhibit the most prolific authors in functional ingredients. It has illustrated that “Barros L” and “Ferreira ICFR” are the most prominent authors in this field, with ten publications each. The most prominent authors, Barros L, 2022 and Ferreira ICFR, 2022 investigated wild edible mushrooms and concluded that they can be a source of nutritional and functional components. Hence, wild edible mushrooms can be incorporated into a balanced diet as a source of proteins and can be utilized for innovative bio-based formulation. Frias J, 2020; Martínez-Villaluenga C, 2020 studied barley grain (flour) and found the ideal method of germinating grain for 3.5 days, maintaining the temperature at 16°C.for producing nutrient-rich and functional barley flour. It is worth emphasizing that these articles have studied various foods like rosemary, xoconostle fruit (opuntia mature scheinvar cv. Rosa) by-products, tarragon, and pineapple by-products for their phenolic and bioactive compounds as well as techniques like fermentation, ultra-sonication, and biochemical and molecular profiling. Table 6 displays a summary of foods investigated by prominent authors.

**Table 4.** Most prolific authors.

Author	Year	Title	Source	DOI	Total Citation	Total Citation/Year
BARROS L	2016	" *Rosemary extracts in functional foods: extraction, chemical characterization and incorporation of free and microencapsulated forms in cottage cheese.”*	FOOD AND FUNCTION	10.1039/c6fo00270f	54	6.75
BARROS L	2017	*“Coloring attributes of betalains: a key emphasis on stability and future applications.”*	FOOD AND FUNCTION	10.1039/c7fo00144d	45	6.429
BARROS L	2016	*“Non-fermented and fermented jabuticaba (myrciaria cauliflora mart.) Pomaces as valuable sources of functional ingredients.”*	FOOD CHEMISTRY	10.1016/j.foodchem.2016.04.011	41	5.125
BARROS L	2015	*“Xoconostle fruit (opuntia matudae scheinvar cv. Rosa) by-products as potential functional ingredients.”*	FOOD CHEMISTRY	10.1016/j.foodchem.2015.04.012	32	3.556
BARROS L	2018	*“Edible flowers of tagetes erecta l. As functional ingredients: phenolic composition, antioxidant and protective effects on caenorhabditis elegans.”*	NUTRIENTS	10.3390/nu10122002	28	4.667
BARROS L	2016	*“Ceratonia siliqua l. Hydroethanolic extract obtained by ultrasonication: antioxidant activity, phenolic compounds profile and effects in yogurts functionalized with their free and microencapsulated forms.”*	FOOD AND FUNCTION	10.1039/c6fo00100a	18	2.25
BARROS L	2020	*“Potential health claims of durum and bread wheat flours as functional ingredients”*	NUTRIENTS	10.3390/nu12020504		4.25
BARROS L	2016	*“Tarragon phenolic extract as a functional ingredient for pizza dough: comparative performance with ascorbic acid (e300).”*	JOURNAL OF FUNCTIONAL FOODS	10.1016/j.jff.2016.08.019	8	1
BARROS L	2022	*“Biochemical and molecular profiling of wild edible mushrooms from huila, angola.”*	FOODS	10.3390/foods11203240	0	0
BARROS L	2022	*“Pineapple by-products as a source of bioactive compounds with potential for industrial food application.”*	FOOD AND FUNCTION	10.1039/d2fo00657j	0	0
BRENNAN CS	2018	*“Gluten-free bakery and pasta products: prevalence and quality improvement.”*	INTERNATIONAL JOURNAL OF FOOD SCIENCE AND TECHNOLOGY	10.1111/ijfs.13505	91	15.167
BRENNAN CS	2019	*“Utilisation of beef lung protein powder as a functional ingredient to enhance protein and iron content of fresh pasta.”*	INTERNATIONAL JOURNAL OF FOOD SCIENCE AND TECHNOLOGY	10.1111/ijfs.13927	13	2.6
BRENNAN CS	2016	*“Synergistic effects of barley, oat and legume material on physicochemical and glycemic properties of extruded cereal breakfast products.”*	JOURNAL OF FOOD PROCESSING AND PRESERVATION	10.1111/jfpp.12617	10	1.25
BRENNAN CS	2022	*“Potential applications of hemp (cannabis sativa l.) Extracts and their phytochemicals as functional ingredients in food and medicinal supplements: a narrative review”*	INTERNATIONAL JOURNAL OF FOOD SCIENCE AND TECHNOLOGY	10.1111/ijfs.16116	0	0
CHEN J	2019	*“Structure, physicochemical properties and adsorption function of insoluble dietary fiber from ginseng residue: a potential functional ingredient.”*	FOOD CHEMISTRY	10.1016/j.foodchem.2019.01.114	94	18.8
CHEN J	2016	*“Chemical components of cold pressed kernel oils from different torreya grandis cultivars.”*	FOOD CHEMISTRY	10.1016/j.foodchem.2016.04.053	52	6.5
CHEN J	2021	*“Protein-polyphenol functional ingredients: the foaming properties of lactoferrin are enhanced by forming complexes with procyanidin.”*	FOOD CHEMISTRY	10.1016/j.foodchem.2020.128145	51	17
CHEN J	2020	*“Amino acid-amidated pectin: preparation and characterization.”*	FOOD CHEMISTRY	10.1016/j.foodchem.2019.125768	20	5
FERREIRA ICFR	2016	*“Rosemary extracts in functional foods: extraction, chemical characterization and incorporation of free and microencapsulated forms in cottage cheese.”*	FOOD AND FUNCTION	10.1039/c6fo00270f	54	6.75
FERREIRA ICFR	2017	*“Coloring attributes of betalains: a key emphasis on stability and future applications.”*	FOOD AND FUNCTION	10.1039/c7fo00144d	45	6.429
FERREIRA ICFR	2016	*“Non-fermented and fermented jabuticaba (myrciaria cauliflora mart.) Pomaces as valuable sources of functional ingredients.”*	FOOD CHEMISTRY	10.1016/j.foodchem.2016.04.011	41	5.125
FERREIRA ICFR	2015	*“Xoconostle fruit (opuntia matudae scheinvar cv. Rosa) by-products as potential functional ingredients.”*	FOOD CHEMISTRY	10.1016/j.foodchem.2015.04.012	32	3.556
FERREIRA ICFR	2018	*“Edible flowers of tagetes erecta l. As functional ingredients: phenolic composition, antioxidant and protective effects on caenorhabditis elegans.”*	NUTRIENTS	10.3390/nu10122002	28	4.667
FERREIRA ICFR	2016	*“Ceratonia siliqua l. The hydroethanolic extract obtained by ultrasonication: antioxidant activity, phenolic compounds profile and effects in yogurts functionalized with their free and microencapsulated forms.”*	FOOD AND FUNCTION	10.1039/c6fo00100a	18	2.25
FERREIRA ICFR	2020	*“Potential health claims of durum and bread wheat flours as functional ingredients.”*	NUTRIENTS	10.3390/nu12020504	17	4.25
FERREIRA ICFR	2016	*“Tarragon phenolic extract as a functional ingredient for pizza dough: comparative performance with ascorbic acid (e300).”*	JOURNAL OF FUNCTIONAL FOODS	10.1016/j.jff.2016.08.019	8	1
FERREIRA ICFR	2022	*“Biochemical and molecular profiling of wild edible mushrooms from huila, angola.”*	FOODS	10.3390/foods11203240	0	0
FERREIRA ICFR	2022	*“Pineapple by-products as a source of bioactive compounds with potential for industrial food application.”*	FOOD AND FUNCTION	10.1039/d2fo00657j	0	0

**Table 5.** Food studied by most influential articles.

Food source	Functional Ingredients	Result	Reference
Sumac	Phenolic compounds	HPLC-DAD/QTOF-MS is a highly efficient method of analyzing phenolic and other phytochemicals in R. coriaria L (Sumac). By using this technique, a wide range of 211 compounds were tentatively identified in Sumac	Abu-Reidah, 2014
Peanuts	Proteins, 20 Amino Acids, Bioactive compounds like arginine (USDA,2014), Resveratrol, Phytosterols, Fiber, calcium, low on glycemic index, Phenolic acids and flavonoids,	Peanuts are a great source of nutrition. They can be abundantly utilized, especially in a country like India which is one of the leading producers of peanut	Arya, 2007
Microalgae	Lipids, Microalgal proteins,bioactive peptides,caretinoids(b-carotene),essential vitamins (A, B1, B2, B6, B12, C, E, nicotinate, biotin, folic acid and pantothenic acid)	Microalgae have the potential to provide a reliable supply of numerous valuable natural compounds such as pigments, PUFAs, carbohydrates, and proteins that are widely used as functional ingredients. However, there are still several significant obstacles to overcome before eukaryotic microalgae and cyanobacteria can become widely used as food commodities instead of remaining in a niche market.	Buono, 2014
Lamarin from Brown Algae	Dietary Fiber, Biofunctional activities, bioactive compounds such as polysaccharides, peptides, omega-3 fatty acids, carotenoids, phenolics, vitamins, and minerals	The lamarin can be obtained by using water and a slightly acidic environment. To enhance the amount and speed of extraction use of microwaves, ultrasonic waves, and supercritical fluids could be explored. Using laminarin or laminarin-rich extracts as functional food ingredients requires further investigation.	Kadam, 2014
Microalgae	Bioactive compounds, such as vitamins, essential amino acids, polyunsaturated fatty acids, minerals, carotenoids, enzymes, and fibre, Omega3 Polyunsaturated Fatty Acids (ω-3 PUFAs),caretonoid	Microalgae, show great potential as a valuable source of functional food products, which can augment the nutritional value of various foods. This is because of their balanced chemical composition and fast rate of growth.	Matos, 2017
Mango	Dietary Fiber	Products with balanced components and low predicted glycemic index response	Quiros-Sauceda et al., 2014
Extruded wheat bran	Dietary Fiber	An increase in Dietary fiber content and lower glycemic index was achieved.	
Oat and wheat	Dietary Fiber	The cupcakes' quality characteristics were enhanced by incorporating 30% dietary fiber.	
Mango Peel	Dietary fiber with associated phenolic compounds	Dietary fiber and polyphenols content increased by 14% and 90%, respectively	
The by-product of apple juice	Dietary fiber with associated phenolic compounds	Increase the dietary fiber and polyphenols content to 14% and 7.16 mg g1, respectivel	
Wine grape pomace	Dietary fiber with associated phenolic compounds	Increase dietary fiber and total phenolic content, also delay lipid oxidation of samples during refrigeration storage.	
Corn maize	Gallic acid-loaded zein fiber	In this study, researchers could effectively add Gallic acid to zein sub-micron fibers using electrospinning at varying ratios. These findings suggest that the resulting fiber mats could be used in packaging materials and as scaffolding for microbial cultures.	Neo, 2013
Banana and banana peel	Phenoloc compounds-gallic acid, catechin, epicatechin, tannins, and anthocyanin. Flavonols- quercetin, myricetin, kaempferol and cyanidin. Carotenoids, Phytosterols,Antioxidant activity	Banana varieties with high levels of bioactive compounds should be identified, promoted, and used in breeding programs to create bio-fortified cultivars. The peel of bananas is also a rich source of bioactive compounds and dietary fiber and could be used as a functional food source.	Singh, 2016
Brown seaweeds, brown algae	Fucoidans-	Studies have shown that fucoidans have a significant impact on human health and nutrition due to their many biological activities and health benefits, and functional uses in areas such as pharmaceuticals, nutraceuticals, cosmeceuticals, and functional foods.	Thanh-Sang Vo, 2013
Food ingredients	Polyphenols, carotenoids, vitamins, enzymes, and probiotics	Starch-based systems have effectively encapsulated food ingredients with a high level of efficiency. When encapsulation is successful, it can improve the overall quality of food products	Zhu, 2017

**Table 6.** Food studied by top 10 prolific authors.

Foods	Functional Ingredients	Result	Reference	
Wild edible mushrooms (WEM)	Carbohydrates, proteins, and ashes also present low amounts of fat, Mannitol (sugar), organic acids, phenolic acids, and hydroethanolic extracts, responsible for their antioxidant, antibacterial, and antifungal activities.	Our inquiry adds to the recognition and comprehension of WEMs as crucial supplementary food sources in Angola, with some being documented for the first time. This encourages their use as essential nutritional and functional components, which can be incorporated into a balanced diet and utilized in innovative bio-based formulations.	BARROS L, 2022; FERREIRA ICFR, 2022	
Barley grain (flour)	Vitamins B1, B2 and C, proteins,phenolic compounds	The ideal method for producing nutrient-rich and functional barley flours was found to be germination for a period of 3.5 days at a temperature of 16°C. When sprouted under these conditions, the β-glucan content of the barley was maintained at 87% of its initial level, and the sprouts showed levels of ascorbic acid, riboflavin, phenolic compounds, and GABA that were between 1.4 and 2.5 times higher than in non-sprouted grains.	FRIAS J, 2020; MARTÍNEZ-VILLALUENGA C, 2020	
Whey protein	Antioxidants, emulsifiers, and foaming agents	Comprehending the diverse forms of protein-polyphenol interactions is crucial to create new whey protein-polyphenol components that can be used for particular purposes in food systems.	MCCLEMENTS DJ, 2021	
Carob pulp	Bioactive extracts-Antioxidants	This research demonstrated that microencapsulation is effective in preserving the functional components in food products, while also maintaining the structure of polyphenols extracted from carob pulp. Additionally, using microencapsulation led to an improvement in the antioxidant strength of the end product.	SANTOS-BUELGA C, 2016	
Capparis spinosa L. (C. spinosa)	Flavonoids, phenolic acids, alkaloids, volatile oils, fatty acids, and polysaccharides	This article systematically reviews the botanical characteristics, traditional edible uses, phytochemical composition, bioactivities, and safety of C. spinosa. Additionally, it emphasizes the potential uses of C. spinosa in foods, which is being highlighted for the first time	WANG Y, 2022
Edible hemp (Cannabis sativa or Industrial hemp)	Phytochemicals, functional metabolites, such as tetrahydrocannabinol (THC), cannabidiol (CBD), and other cannabinoids	This comprehensive review examines the primary phytochemicals found in hemp and explores the key obstacles when using these substances in food and pharmaceutical products. The challenges include stability, toxicity, legal restrictions, and the isolation, extraction, and purification of these compounds.	BRENNAN CS, 2022
Food ingredients	Amino acids- Amino acid-amidated pectin (AAAP)	The AAAP conjugates have the potential to be appropriate as novel functional components in the food sector.	CHEN J, 2022
Food ingredients	Apigenin (APG)	The use of APG-OMT CM has the potential to be highly effective in functional food applications. Significantly, the research indicates a promising method of delivering hydrophobic food ingredients, which could improve their bioavailability.	LI B, 2021

### 3.6 Recent articles on functional biscuits

Table 7 summarizes articles from 2021-2023 that investigate foods for their potential use in developing functional biscuits. Recent studies have examined the potential of Amla and Apple pomace, Thyme, Tannat grape skin, Jujub flour, Bee pollen, Chickpea, Lemon peel, and lemon pomace to add functionality to new biscuits. The studies have demonstrated positive results regarding the acceptability and quality of cookies/biscuits in terms of functionality.

**Table 7.** Foods used by recent studies to develop functional biscuits.

Functional Ingredients	Food source	Result	Reference
Functional property	Amla and apple pomace	The replacement of apple and amla pomace up to 10% for biscuit preparation was desirable. The inclusion of amla, apple, and pomace mix in the biscuits improves the nutritional content, functional properties, and overall qualities of the product.	Patel, 2022
Dietary fiber, phenolic content, antioxidant activity	Corncob (Zea Mays L.)	Enzymatic treatment of products made them less hard and more easily breakable while increasing their overall acceptability compared to products made without such treatment. This serves as an example of how corncob can be processed using enzymes to create a high-fiber supplement for human consumption that is readily available.	Hoang, 2022
Nutritional benefits	Thyme (Thymus vulgaris L.)	Thyme leaf powder cookies containing 3% TLP were found to be the most accepted in terms of their sensory qualities. This research not only confirms the nutritional value and taste evaluation of cookies made with thyme leaf powder but also guides the wider community on the importance of incorporating thyme herbs in their diets.	Waheed M, 2022
Flavonoids, phenolic acids, and anthocyanins, dietary fiber	Tannat Grape Skin	achieved a sensory score of 5.1 for biscuits. Yogurt and cookies were made by adding Tannat grape skin, and they have nutritional benefits such as being "no-added sugars" and a "source of fiber". These products may have the potential to influence important biochemical processes related to the development of diabetes.	Fernández-Fernández A. M, 2022
Nutritional benefits	Cassava starch (Manihot esculenta)	The study evaluated the quality of cookies made using microwave-modified pulp starch by replacing 10-40% of wheat flour. The cookies were deemed acceptable with a sensory score of above 5. This research could lead to the identification of functional ingredients that offer health benefits beyond just nutrition.	Khurshida S, 2022
Antioxidant activity, fiber and protein content	Tamarindus indica seed	The utilization of T. indica seed flour enhanced the fiber content of biscuits significantly up to 3.88 ± 0.02% and protein content up to 11.22 ± 0.05 (p < 0.05). The sensory and antioxidative properties of T. indica seeds were improved with moderate roasting, while microwave roasting was an effective method for roasting the seeds.	Bolek S, 2021
Dietary fiber, ash and total phenolic contents	Jujube flour (JF) or jujube fiber concentrate (JFC)	The weight, thickness, diameter, and spread ratio of enriched biscuits showed no differences. The sensory quality of the biscuits was acceptable, regardless of the JF level. A maximum of 10% of JFC can be added to achieve the desired quality of biscuits.	Masmoudi M, 2021
Phenolic content and antioxidative activity	Bee pollen	Bee Pollen is a component that can potentially enhance both the quality standards and functional characteristics of cookies.	Dundar A.N,2021
Dietary proteins (17–22%)	Chickpea (Cicer arietinum L.)	Chickpea proteins and hydrolysates show potential as functional ingredients and may become a viable substitute for a variety of applications (like cookies) due to the lack of strong flavor, neutral taste, and pale color	Boukid F,2021
Phenolic content and antioxidant activity	Lemon peel and lemon pomace	From a sensory perspective, the biscuits were visually appealing, flavorful, and aromatic. In summary, the results suggest that incorporating lemon processing by-products resulted in biscuits that were not only delicious but also beneficial for health.	Imeneo,2021

### 3.7 Science mapping

To understand a particular research subject through visual depiction is conducted by using science-mapping analysis. This evaluation involves several types of analysis, including thematic, factorial, temporal, and network analysis. The study has utilized Network analysis to elucidate the primary themes concerning functional ingredients research.

### 3.8 Network analysis

Network analysis was conducted by applying co-authorship mapping and co-occurrence mapping using VOS viewer software.

To examine the co-authorship link minimum of 10 authors with a max 75-citation threshold were selected. Out of 396 authors, 30 fulfilled the criteria. The mapping demonstrated that all top authors with the highest citation of 331 (red cluster) and lowest citation of 77 (grey cluster) had zero total link strength among themselves.

During co-occurrence mapping, all keywords that were ministered by the full counting method were considered as part of the analysis. To enhance accuracy, the study implemented certain parameters to perform analysis to avoid repetition of keywords. Additionally, the lowest of five instances were set to select the keywords. As a result, out of 1149 keywords, 32 satisfied multiple criteria after data cleaning.

**Figure 6.**
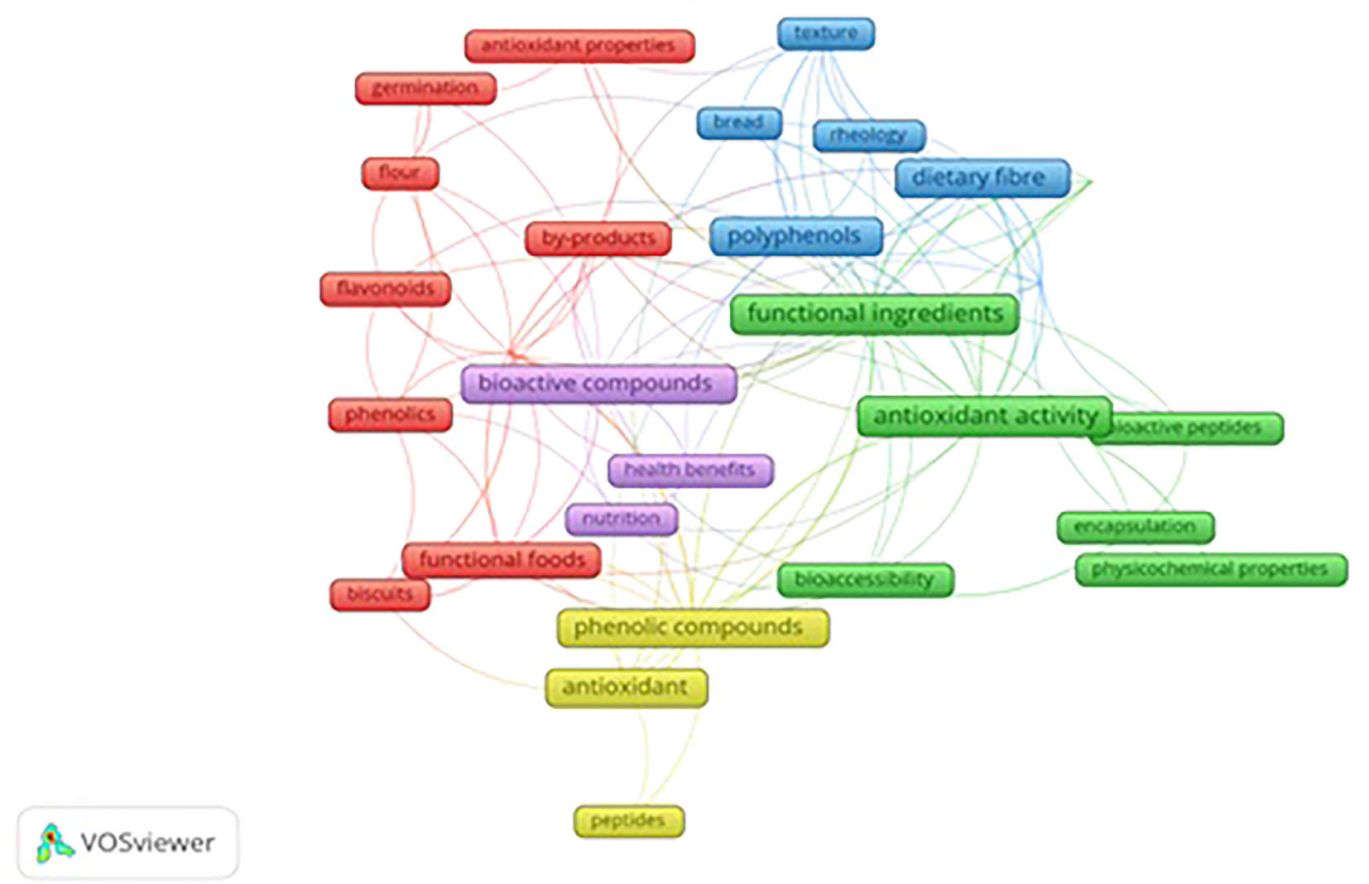
Network Visualization of Keywords.

The clustering technique is used to focus on three clusters out of five clusters. Figure 6 demonstrates the network visualization that appeared through scientific articles. The cloud diagram illustrates the frequency of a word in the article and its correlation with other keywords. Each term is depicted as a frame in the diagram, and the size of the frame represents the number of times the term appears in publications. Different colors represent the frames grouped into clusters. The curved lines indicate the proximity of the terms, while the thickness of the lines reflects the strength of the connection between pairs of topic areas or keywords. The clusters provide insight into the relationship between different topics.

Three clusters out of five, red, green, and blue, are more prominent than the rest. The red areas represent topics related to antioxidant properties, by-products of food processing, developing functional foods, biscuits, and techniques. The network analysis showed a strong interdependence of these concepts like food processing byproducts, using functional foods like biscuits with antioxidant properties by following specific techniques or processes. Techniques and food processing byproducts could act as bridges to transform food waste into healthy food items particularly widely consumed biscuits.

The green clusters comprise functional ingredients, antioxidant activity, bioactive peptides, bioaccessibility, encapsulation, and physiochemical properties. Among the repeatedly occurring keywords, antioxidant activity tops the list with 33 occurrences. The green cluster focuses on functional ingredients with other terms reflecting the ways to measure their performance (antioxidant activity) and ensure their absorption by the body (bioaccessibility) through technique (encapsulation).

The blue cluster highlights functional properties, dietary fiber, rheology, polyphenols, texture, and bread. This cluster shows the interplay between ingredients (dietary fiber and polyphenol) their functional effects (rheology and functional properties) and the quality outcome of the final product (bread).

The network analysis of keywords implies that research on functional ingredients is multifaceted. It highlights the deep understanding of how functional ingredients interact with other foods and influence the overall quality and texture of developed food products.

The software displays the keywords that have the most frequent occurrences alongside other keywords displayed in Table 8. “Occurrence” refers to the number of articles that feature the keyword. Antioxidant activity, functional ingredients, Antioxidants, Dietary fiber, Phenolic compounds, Bioactive compounds, Polyphenols, Antioxidant capacity, Functional properties, and Functional foods are the most highly co-occurring keywords with occurrence weights (total link strength) as 33(25), 31(22), 23(12), 20(21), 20(21), 18(19), 18(20), 17(20), 14(13), 13(13).

**Table 8.** Keywords and occurrence link.

Keywords	Cluster number	Link	Occurrence	Total link strength
Antioxidant activity	Cluster 2 (green)	15	33	25
Functional ingredients	Cluster 2 (green)	13	31	22
Antioxidant	Cluster 4 (yellow)	8	23	12
Dietary fibre	Cluster 3 (blue)	10	20	21
Phenolic compounds	Cluster 4 (Yellow)	10	20	21
Bioactive compounds	Cluster 5 (Purple)	15	18	19
Polyphenols	Cluster 3 (blue)	14	18	20
Antioxidant capacity	Cluster 1 (red)	13	17	20
Functional properties	Cluster 3 (blue)	10	14	13
Functional foods	Cluster 1 (red)	9	13	13

**Figure 7.**
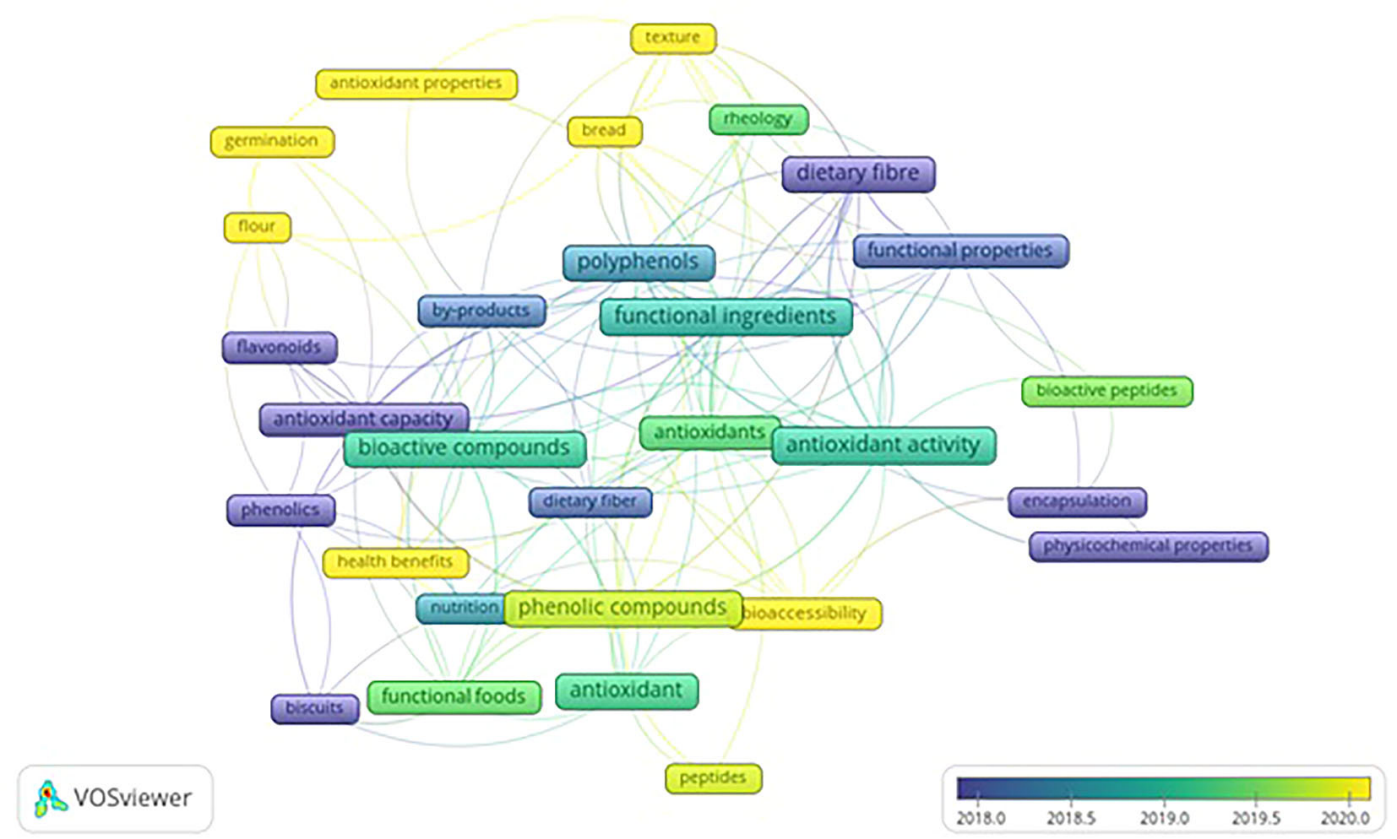
Overlay Visualization.

**Figure 8.**
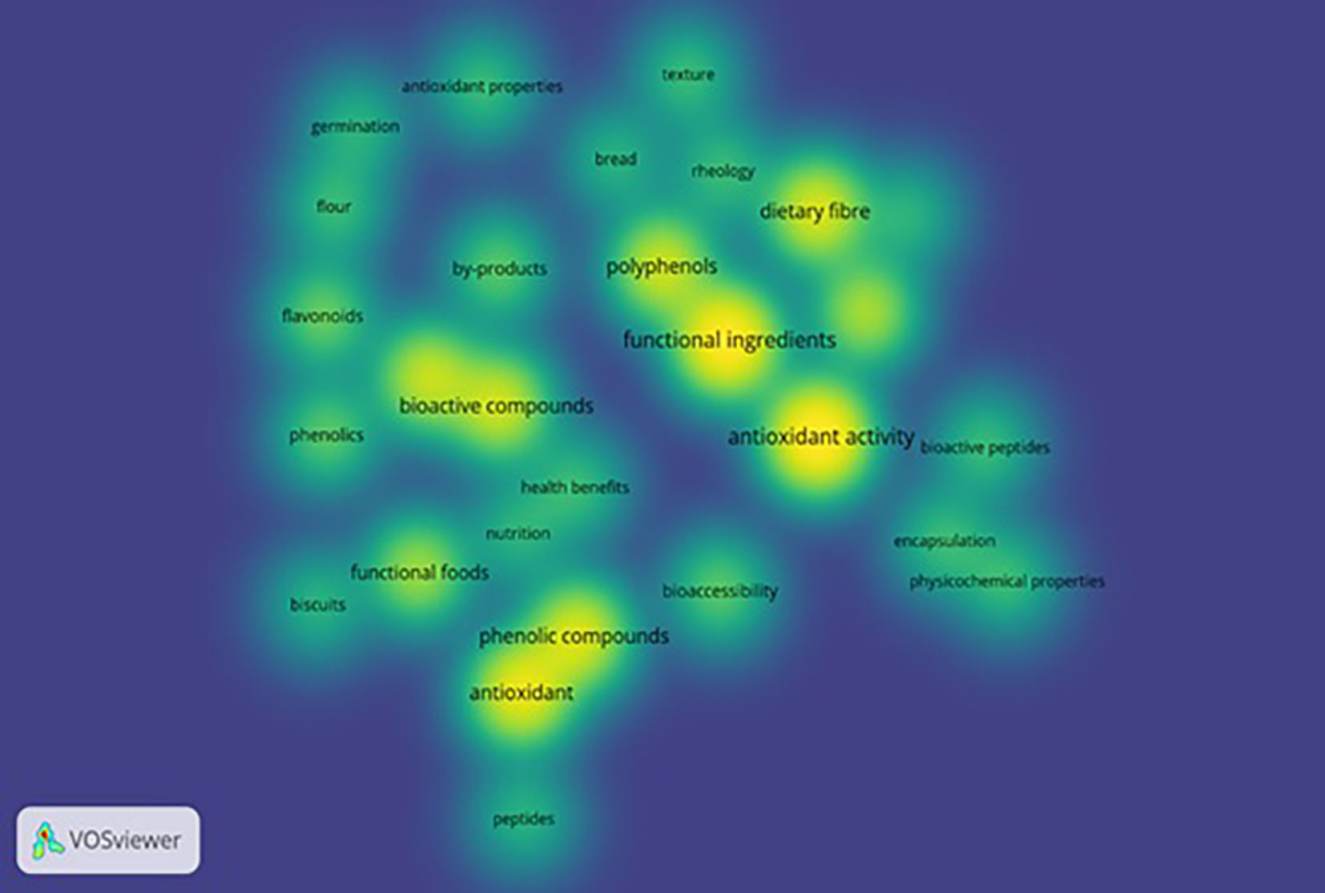
Density Visualization.

VOS viewer can show three different mapping visualizations which are created from Figure 6 (network visualization), Figure 7 (overlay visualization), and Figure 8 (density visualization).

### 3.9 Overlay visualization

Figure 7 displays developments related to keywords over the researched periods. The colors of the keyword frames depict the researched period. The yellow cluster represents the keywords from studies published from 2020 onwards. Antioxidant activities, health benefits, and bioaccessibility are observed as the most occurred keywords in recent studies, including germination, bread, texture, and flour.

### 3.10 Density visualization

The depth of researched areas can be seen in Figure 8. Deep concentration of colors interprets topics undertaken by more researchers to study. The most deliberated topics by researchers are functional ingredients, antioxidant activity, bioactive compounds, phenolic compounds, antioxidants, polyphenols, dietary fiber, functional foods, flavonoids, phenols, and bioaccessibility.

## 4. Discussion

This bibliometric analysis retrieved articles from Scopus and effectively examined the publication trends in functional ingredients to derive insight into their usefulness in developing biscuits with functional properties. The results corroborated previous studies that have demonstrated biscuits as an effective medium for incorporating functional ingredients. ^4^^,^^6^ A bibliometric analysis approach also elucidated current trends, evolution, and potential for future advances in functional ingredient research from a global perspective.

### 4.1 Publication trends ( Figures 3 & 4)

Although the selected publications’ issue year is 2013, the publication trend gained momentum from 2015. This trend could be associated with the United Nations’ (2015b) seventeen sustainable development goals (SDGs) in the 2030 agenda for sustainable development, adopted by United Nations Member States in 2015. The increase in research publications on functional ingredients may be attributed to the SDG goals, specifically the second goal of “Zero Hunger” and the third goal of “Good Health and Well-being”. ^18^ The year 2022, with 76 publications and an annual growth of 39.5%, demonstrates the increasing significance of functional ingredient research worldwide.

### 4.2 The most influential article ( Table 2)

“HPLC–DAD–ESI-MS/MS screening of bioactive components from Rhus coriaria L. (Sumac) fruits” emerges as the most contributing article with 331 citations. ^2^ Followed by “Encapsulation of food grade antioxidant in natural biopolymer by electrospinning technique: A physicochemical study based on the zein-gallic acid system” ^10^ at 288 citations, sourced from Food Chemistry. Notably, both articles investigated techniques to extract bioactive compounds (211) from sumac and innovative gallic acid combined zein sub-micron fiber mat (packaging material) with antioxidant activity through an electrospinning technique, respectively. Both articles emphasize the significance of technology and techniques for extracting bioactive compounds from foods to develop functional materials for the packaging industry. Similarly, Ref. 19 study examines the utilization of microencapsulation, vacuum impregnation, and nutrigenomic techniques in developing functional foods. It underscores the importance of technologies to prevent the degradation of bioactive compounds. ^4^ Although the study was initiated focusing on the efficacy of functional ingredients in developing functional biscuits, the most influential article lacks the focus of the study, which could be considered as a limitation of the study as well as a gap in the rarity of studies explicitly undertaken in functional ingredients to develop functional biscuits. This limitation could have severe implication on innovation, technology and knowledge for future processes of developing functional biscuits. Hence, the present study could be considered unique and a step forward to bridge the gap.

### 4.3 Most Contributing Journal ( Table 3)

The results indicate that “Food Chemistry” and “Food & Function” were among the most contributing journals, with 97 and 40 articles, respectively. Both articles achieved an “h” index of 39 & 19 respectively.

### 4.4 Relevant authors ( Figure 5)

Among the most relevant authors, Barros L and Ferreira ICFR account for ten publications in food ingredients research which causes the expansion in research. The co-authorship was examined by using network analysis using VOS viewer software. Among the most cited 30 authors, zero total link strength was detected, indicating that authors have not collaborated in analyzed papers.

### 4.5 Food investigated by influential articles ( Table 5)

The most influential articles examined foods such as sumac, ^2^ peanuts, ^6^ brown algae, ^20^ microalgae, ^10^ extruded wheat bran, ^5^ and mango. ^21^^,^^22^ These studies on various foods provide opportunities for developing novel functional biscuits and validate the assertion made by Ref. 4 that foods with functional properties represent a potential for creating innovative foods that could meet current demand. The successful production of functional biscuits is evidenced in other studies conducted by Ref. 23 on soy flour, rice bran, ^24^ industry by-products like hemp, ^25^ artichoke by-products, ^26^^,^^27^ Finger Millet ^28^ and Carrot Pomace. ^28^^,^^29^ Correspondingly, a study by Ref. 8 highlights the finding and states that the substantial increase in the popularity of readily consumable functional foods in recent years is motivating manufacturers to create novel, nutrient-rich foods that offer health benefits. ^8^ Moreover, the data shows that the recent studies conducted from 2020 onwards have explored foods like industrial waste (Apple and Amla, Corncob, and Thyme), as well as industry by-products (upcycled defatted sunflower seed flour) for developing functional biscuits, validating the answers to framed research questions 1-4.

### 4.6 Most researched functional ingredients ( Table 8)

The review examines the status and trends in functional ingredient research. The network analysis reveals five clusters: “Functional ingredients,” “Antioxidant Activity,” “Bioactive compound,” “Phenolic compounds,” and “Dietary Fiber.” Among the keywords, “Antioxidant activity” is most prevalent with 33 occurrences, which corroborates the findings of a study by Ref. 30, reporting “antioxidant activity” with 57 occurrences. ^30^ The second most frequent keyword is “Functional ingredient,” with 31 occurrences, indicating a growing trend toward considering the relationship between food and health. A study ^2^ demonstrates the association between dietary fiber and phenolic compounds, asserting that incorporating phenolic compounds and dietary fiber in food components not only enhances the performance of foods but is also utilized to develop functional foods that offer health benefits. ^2^ Recent publications have focused their studies on “Bioaccessibility”, “Antioxidant properties”, “Health benefits”, “Flour”, “Germination”, “Bread”, and “Texture”. The study by Ref. 30 concludes that a novel approach is necessary to evaluate bioaccessibility and bioavailability by investigating the efficacy of leafy Phenolic compounds for access and utilization in commercial applications. ^30^ Publications by Barros L (2022), Ferreira ICFR (2022), and Mc Clements DJ (2021) have assessed the association between natural food sources’ antioxidant properties and health benefits. The findings by Faller (2023) and Brito (2021) have enumerated health benefits such as Antidiabetic, Anticancer, Hepatoprotective, Neuroprotective, Anti-inflammatory, Anti-arthritic, Antibacterial, Insecticidal effects, and potential impacts on Cardiovascular diseases, Obesity, Diabetes, and Cancer—the rational narrated answers to research question 5.

### 4.7 Upcoming study trends

The integration of functional ingredients in developing novel functional foods is increasing as studies have successfully incorporated various functional ingredients such as phenolic compounds, bioactive compounds, flavonoids, and antioxidants into functional foods specific to bakery. Although consumers perceive baked foods as unhealthy, the publication data shows numerous research studies in recent decades have aimed at improving the nutritional content of biscuits. The results of these studies demonstrate an increase in technological and technique-oriented innovations in the context of extracting functional ingredients from existing foods. Traditional techniques such as fermentation, germination, and sprouting are gaining prominence in recent studies to extract functional ingredients from grains and pulses. Furthermore, the impact of technologies such as encapsulation, freeze-drying, and electrospinning requires further investigation to assess their effect on developed functional foods, as highlighted through the evaluated articles. Food packaging material with the added advantages of functionality, lower production costs, and increased shelf life represents an emerging field of study that necessitates scientific attention. Future research is required to assess the potential of peptides, nutrition, and germination due to their status of limited network connectivity. Additionally, the study suggests that further investigations are necessary to enhance the bioaccessibility and bioavailability of bioactive compounds from the consumption of developed functional foods. From the perspective of developing functional biscuits, future studies could explore the technological advancements to extract functional ingredients and evaluate the bioaccessibility and bioavailability through the consumption of functional biscuits.

## 5. Conclusion

The study employs bibliometric analysis to examine the functional ingredients and biscuits literature. The investigation assessed 395 documents and aspects such as variations in the number of publications over the research period, articles published, sources of publications, prolific authors, and keywords utilizing R studio and VOS viewer. Analysis indicates that the number of publications related to the topic will increase over time. Figures 6- 8 present mapping derivations based on title, abstract, keyword, and publications using VOS viewer. The study used search keywords “functional ingredients,” “functional biscuits,” “functional ingredients,” “functional biscuits,” and “nutritional biscuits,” but only 10 % of total publications are focused on examining the functional properties of nutritionally enhanced biscuits/cookies. Hence, the study highlights the need and opportunity for future studies focusing on developing functional biscuits by using advanced techniques for extracting functional ingredients. Additionally, the insight gathered from the study provides a means to understand the efficacy of the functional ingredients in developing functional biscuits, as the data proves the successful incorporation of functional ingredients by using foods with functional properties. Additionally, the study demonstrates that functional ingredients are associated with various other fields through the application of visualization mapping (VOS viewer), which includes absorption of functional ingredients and development of packaging material. The study findings highlight that keywords without network connections to other keywords, including peptides, germination, and nutrition, can be potential topics for future research. The experiment study done by many publications with various techniques like fermentation, germination, and freeze-drying provides an opportunity for future research to study their applicability to develop new formulations of functional biscuits. Among the publication data, bread was investigated for its flour replacement without compromising texture, emphasizes that textural evaluation of novel foods could be a prominent area for future studies. Hence, the study recommends that future studies focused on developing functional biscuits would boost the biscuit industry to heights as such studies would provide the foundation for biscuit manufacturers to introduce innovative functional biscuits in the market.

Nevertheless, a limitation of the study is that data was generated solely from the Scopus database, and future studies could utilize various other sources such as the Web of Science or PUB Med. Additionally, data was retrieved from Scopus on 17 ^th^ February 2023, which could be considered a limitation as the data’s comprehensiveness could be questioned. Results might vary if the study is replicated with a more elaborate timeline.

## Data Availability

No data are associated with this article.
