# Supplementary material for: A bibliometric review of functional ingredients and their efficacy in developing functional biscuits
Source: F1000Res. 2024 Dec 16;13:421. Originally published 2024 Apr 29. [Version 3] doi: 10.12688/f1000research.148029.3 (PMC11754956; doi:10.12688/f1000research.148029.3)
Supplement: Peer review history

# Reviewer report: Reviewer response for version 3

**DOI:** 10.5256/f1000research.175245.r352826

**Published:** 2025-01-29

**Authors:** Smriti Gaur

**Affiliations:** 1 Jaypee Institute of Information Technology, Noida, Uttar Pradesh, India

**Overall Comments:** The authors presented an article, “ *A bibliometric review of functional ingredients and their efficacy in developing functional biscuits*”. While the study provides valuable insights into the trends, contributions, and applications of functional ingredients in biscuit development, there are several areas where improvements can enhance the clarity and overall presentation. At some places, the language precision can also strengthen the manuscript’s readability. I have mentioned few specific comments, addressing these comments which will significantly improve the quality and impact of the manuscript.

**Specific Comments:**

1. The search strategy mentions alternate terminologies like "enriched biscuits" and "functional cookies," but it is unclear whether all relevant synonyms were comprehensively considered or not. The authors must clarify how they ensured the inclusion of all potential variations or related terms to avoid missing significant studies,
2. The sentence " *The search thread followed the title, abstract, and keywords to identify documents*" could be more precise to- " *The search was conducted by filtering titles, abstracts, and keywords to identify relevant documents.*".
3. The phrase " *The publication of articles increased close to double during the years 2021&2022*" could be rephrased to- " *The number of articles published nearly doubled between 2021 and 2022.*".
4. Consider adding a brief introductory sentence to explain the significance of Table 2 before diving into the details.
5. Phrases like " *is the most prominent study*" and " *secured the second spot*" could be replaced with more formal alternatives such as " *is the most cited study*" or " *ranked second in total citations*."
6. The sentence " *Interestingly both journals have amassed total citations '4144' and '1329' and stand at '39' and '19' h index individually with their higher contributions*" is overly complex- Kindly rephrase the sentence to deliver a more formal tone.
7. When discussing recent studies (Table 7), ensure consistent formatting for food names and methods. For instance, avoid switching between common and scientific names without context.
8. Kindly elaborate on how data cleaning for keywords was performed?
9. The limitation of using only the Scopus database is noted. Consider suggesting how incorporating additional databases, such as Web of Science or PubMed, might broaden the scope and validity of the findings.
10. Abbreviations should be placed after full names in the first place appearing throughout the manuscript (abstract and text separately).
11. The manuscript mentions many ingredients, but their scientific names are missing in several places. Kindly include the corresponding scientific names for all ingredients.
12. Scientific names (genus and species) should always be italicized as per convention. Carefully review the manuscript to ensure proper formatting of all scientific names.

Are the rationale for, and objectives of, the Systematic Review clearly stated?

Yes

Is the statistical analysis and its interpretation appropriate?

Yes

If this is a Living Systematic Review, is the ‘living’ method appropriate and is the search schedule clearly defined and justified? (‘Living Systematic Review’ or a variation of this term should be included in the title.)

Not applicable

Are sufficient details of the methods and analysis provided to allow replication by others?

Yes

Are the conclusions drawn adequately supported by the results presented in the review?

Yes

Reviewer Expertise:

Functional food

I confirm that I have read this submission and believe that I have an appropriate level of expertise to confirm that it is of an acceptable scientific standard, however I have significant reservations, as outlined above.

# Reviewer report: Reviewer response for version 3

**DOI:** 10.5256/f1000research.175245.r350126

**Published:** 2025-01-22

**Authors:** Praveen Kumar Dubey

**Affiliations:** 1 School of Agriculture, Lovely Professional University,, Phagwara, India

- The quality of most figures is substandard. Please improve the resolution and presentation of the figures to enhance readability and visual appeal.
- The section on bibliometric tools lacks sufficient detail. Currently, only two software tools are mentioned. Expand this section by including additional tools and providing a comparative discussion of their functionalities and applications.
- Create a separate and detailed discussion on PRISMA and PRISMA-P methodologies. Additionally, compare these frameworks with SPAR-4-SLR, emphasizing how they can be more effective for research on functional foods.
- The number of articles included for bibliometric analysis is not mentioned in Section 2.1.1. Please specify this information to ensure clarity and completeness.
- The section titled *“HPLC-DAD-ESI-MS/MS screening of bioactive components from Rhus coriaria L. (Sumac) fruits”* contains outdated or incomplete citations . Update the references (should be 592) to reflect current and accurate information.
- The citations for the table of contents are missing from the reference section. Ensure that all references related to tables and their contents are included and properly formatted.

Are the rationale for, and objectives of, the Systematic Review clearly stated?

Partly

Is the statistical analysis and its interpretation appropriate?

Partly

If this is a Living Systematic Review, is the ‘living’ method appropriate and is the search schedule clearly defined and justified? (‘Living Systematic Review’ or a variation of this term should be included in the title.)

Yes

Are sufficient details of the methods and analysis provided to allow replication by others?

Partly

Are the conclusions drawn adequately supported by the results presented in the review?

Yes

Reviewer Expertise:

Food Science, non-thermal technology

I confirm that I have read this submission and believe that I have an appropriate level of expertise to confirm that it is of an acceptable scientific standard, however I have significant reservations, as outlined above.

# Reviewer report: Reviewer response for version 3

**DOI:** 10.5256/f1000research.175245.r349196

**Published:** 2025-01-16

**Authors:** Clara Talens

**Affiliations:** 1 AZTI, Food Research, Basque Research and Technology Alliance (BRTA), Bizkaia, Spain

The authors have successfully addressed all the comments and clarified the remaining points.

Are the rationale for, and objectives of, the Systematic Review clearly stated?

Partly

Is the statistical analysis and its interpretation appropriate?

Yes

If this is a Living Systematic Review, is the ‘living’ method appropriate and is the search schedule clearly defined and justified? (‘Living Systematic Review’ or a variation of this term should be included in the title.)

Not applicable

Are sufficient details of the methods and analysis provided to allow replication by others?

Partly

Are the conclusions drawn adequately supported by the results presented in the review?

Partly

Reviewer Expertise:

NA

I confirm that I have read this submission and believe that I have an appropriate level of expertise to confirm that it is of an acceptable scientific standard.

# Reviewer report: Reviewer response for version 2

**DOI:** 10.5256/f1000research.171978.r333818

**Published:** 2024-11-19

**Authors:** Clara Talens

**Affiliations:** 1 AZTI, Food Research, Basque Research and Technology Alliance (BRTA), Bizkaia, Spain

The manuscript has significantly improved in addressing the comments raised in the initial review. The authors have made considerable efforts to clarify their arguments, provide more detailed explanations, and enhance the structure of the paper. However, there are still three **major comments** that require further clarification or revision. Once these aspects are addressed, the manuscript will be in a stronger position for acceptance.

**1. **The justification for choosing the SPAR-4-SLR method over PRISMA is still unclear and requires more precise elaboration. The current statement, *“The PRISMA guidelines were developed for general systematic reviews, and the justification for using guidelines does not support researchers’ decision-making,”* is vague and lacks specificity.

 Authors should explicitly explain what specific advantages SPAR-4-SLR offers for bibliometric reviews compared to PRISMA.

 PRISMA is designed for systematic reviews focusing on evidence synthesis, while SPAR-4-SLR is tailored for reviews aiming to identify research trends, gaps, and patterns, often incorporating bibliometric analysis.

**2.**  The manuscript mentions that the last update of the dataset was conducted on **February 17, 2023**, which raises a limitation regarding the comprehensiveness of the data. The authors should acknowledge this as a limitation in the discussion or conclusion section and, if feasible, provide a rationale for why an update is not possible (e.g., time or resource constraints).

**3.** The section on “most influencing articles” includes a prominent study on polyphenol extraction from Sumac fruits (HPLC-DAD-ESI-MS/MS screening). While this article is highly cited and relevant to functional ingredients, it is not directly related to functional biscuits, which is the core focus of this review. To improve relevance, the authors should prioritize influential articles that discuss the application of functional ingredients in biscuit formulations. If no such articles are prominent, the authors should explicitly state this gap in the literature and discuss its implications for the field.

**Minor comment**:

 In the introduction, the phrase *“products that”* is repeated unnecessarily. This redundancy should be removed to improve readability and flow.

 The manuscript is nearing completion but still requires clarification on methodological justification, acknowledgment of dataset limitations, and refinement of the relevance of influential articles.

Are the rationale for, and objectives of, the Systematic Review clearly stated?

Partly

Is the statistical analysis and its interpretation appropriate?

Yes

If this is a Living Systematic Review, is the ‘living’ method appropriate and is the search schedule clearly defined and justified? (‘Living Systematic Review’ or a variation of this term should be included in the title.)

Not applicable

Are sufficient details of the methods and analysis provided to allow replication by others?

Partly

Are the conclusions drawn adequately supported by the results presented in the review?

Partly

Reviewer Expertise:

food ingredients; food technology; food product development; food science and nutrition; functional foods; systematic literature reviews; data analysis; impact of food ingredients on health

I confirm that I have read this submission and believe that I have an appropriate level of expertise to confirm that it is of an acceptable scientific standard, however I have significant reservations, as outlined above.

# Response

**Published:** 2024-11-24

**Authors:** Varalakshmi Chandra Sekaran

**Affiliations:** Melaka Manipal Medical College, India

**Reviewer’s comments:** **2. Methodology**:

**1. **The justification for choosing the SPAR-4-SLR method over PRISMA is still unclear and requires more precise elaboration. The current statement, *“The PRISMA guidelines were developed for general systematic reviews, and the justification for using guidelines does not support researchers’ decision-making,”* is vague and lacks specificity.
  Authors should explicitly explain what specific advantages SPAR-4-SLR offers for bibliometric reviews compared to PRISMA.

**Response: **The methodology is improved, and the justification is updated.

**Reviewer’s comments: **The manuscript mentions that the last update of the dataset was conducted on **February 17, 2023**, which raises a limitation regarding the comprehensiveness of the data. The authors should acknowledge this as a limitation in the discussion or conclusion section and, if feasible, provide a rationale for why an update is not possible (e.g., time or resource constraints).

**Response: **Added under conclusion section as a limitation.

**Reviewer’s comments: **The section on “most influencing articles” includes a prominent study on polyphenol extraction from Sumac fruits (HPLC-DAD-ESI-MS/MS screening). While this article is highly cited and relevant to functional ingredients, it is not directly related to functional biscuits, which is the core focus of this review. To improve relevance, the authors should prioritize influential articles that discuss the application of functional ingredients in biscuit formulations. If no such articles are prominent, the authors should explicitly state this gap in the literature and discuss its implications for the field.

 Response: Thank you for highlighting the gap. As per instructions, the discussion section is updated.

**Reviewer’s comments: Minor comments:**

 In the introduction, the phrase *“products that”* is repeated unnecessarily. This redundancy should be removed to improve readability and flow.

**Response: **All the comments are looked into, and necessary changes are made to the manuscript.

# Reviewer report: Reviewer response for version 1

**DOI:** 10.5256/f1000research.162293.r305797

**Published:** 2024-08-16

**Authors:** Teti Estiasih

**Affiliations:** 1 Universitas Brawijaya, Malang, East Java, Indonesia

This article provides a bibliographic analysis of the functional ingredients for biscuits. However, the authors can not distinguish well the difference between functional ingredients and bioactive compounds. Functional ingredients are the ingredients used for manufactured foods that either have specific functions such as emulsifying or specific functions on health such as an ingredient high in carotene like carrot. Usually, the authors use the terms of bioactive compounds interchangeably with functional ingredients.  For example: in Tables 3 and 6. The correct term is bioactive compounds instead of functional ingredients. 

 The title of this article is about functional biscuits, however, the analyzed published scholarly articles are general and do not focus on biscuits such as in Tables  2, 3, and 5.

 The authors should mention the sources of the analyzed scholarly articles such as in Figure 3 and the Method section.

 I suggest the Authors revise the title to make it more relevant to the content of this article. I recommend the title be: A Bibliographic Review of bioactive compounds and their efficacy in developing functional foods.

 Lastly, the numbering for cited references should be listed based on the sequence of their citation in the text. For example, the No, 25 should be the No. 1 because this is the first citation.

Are the rationale for, and objectives of, the Systematic Review clearly stated?

Partly

Is the statistical analysis and its interpretation appropriate?

Partly

If this is a Living Systematic Review, is the ‘living’ method appropriate and is the search schedule clearly defined and justified? (‘Living Systematic Review’ or a variation of this term should be included in the title.)

Partly

Are sufficient details of the methods and analysis provided to allow replication by others?

Yes

Are the conclusions drawn adequately supported by the results presented in the review?

Partly

Reviewer Expertise:

Food science and technology

I confirm that I have read this submission and believe that I have an appropriate level of expertise to confirm that it is of an acceptable scientific standard, however I have significant reservations, as outlined above.

# Reviewer report: Reviewer response for version 1

**DOI:** 10.5256/f1000research.162293.r305792

**Published:** 2024-08-10

**Authors:** Clara Talens

**Affiliations:** 1 AZTI, Food Research, Basque Research and Technology Alliance (BRTA), Bizkaia, Spain

**Summary of the manuscript**: The manuscript presents a bibliometric analysis of research on functional ingredients used in developing functional biscuits. It aims to identify current trends, prolific authors, influential articles, and potential future advancements in this research area. The analysis is based on 395 articles retrieved from the Scopus database between 2013 and 2023, employing tools like R Studio and VOS Viewer for performance analysis and science mapping.

**Overall assessment**: The manuscript is well-structured and presents an important analysis of the literature on functional ingredients and their application in biscuits. The methodology is clear, and the use of bibliometric tools is appropriate for the research objectives. However, there are some areas that need improvement to enhance the clarity and impact of the paper.

**Major comments:**

1. **Literature review**:
  - The literature review should be expanded to provide a more comprehensive background on the importance of functional biscuits and their role in health. The current review is somewhat brief and does not adequately establish the context for the bibliometric analysis.
  - There should be more discussion on the different types of functional ingredients commonly used in biscuit formulations and their specific health benefits.
2. **Methodology**:
  - The selection criteria for articles should be explained in more detail. While the use of the SPAR-4-SLR method is mentioned, the manuscript does not clearly describe how the articles were filtered and selected beyond the initial database search. It would be beneficial to include a flowchart illustrating the selection process.
  - The manuscript claims that the SPAR-4-SLR method is superior to PRISMA guidelines but does not provide evidence or references to support this claim. A comparison or justification for this choice would strengthen the methodology section.
3. **Data analysis**:
  - While the results section is thorough, it would benefit from more interpretation of the data. For instance, the manuscript mentions trends in publication growth and the most cited articles but does not discuss why these trends are occurring or their implications for the field.
  - The network analysis section should include a discussion on the significance of the clusters identified and how they relate to the development of functional biscuits.
4. **Discussion and conclusion**:
  - The discussion should be expanded to include the implications of the findings for future research and industry practice. Specifically, how can the identified trends and gaps be addressed in future studies? What are the practical applications of these findings in the food industry?
  - The conclusion section is brief and could be more robust. It should summarize the key findings and suggest actionable recommendations for researchers and practitioners.
5. **Clarity and readability**:
  - Some parts of the manuscript are densely written and could benefit from clearer language and shorter sentences. Simplifying the presentation of the data, particularly in the results section, would make the manuscript more accessible to a broader audience.
  - The use of figures and tables is appropriate, but some could be better integrated into the text. For example, the discussion could reference specific tables or figures more explicitly to guide the reader.

**Minor comments:**

- **Abstract**: The abstract provides a good summary of the paper, but it could be more specific about the key findings and their implications.
- **References**: Ensure all references are current and relevant. Some of the references cited are over a decade old, and it might be beneficial to include more recent studies to support the analysis.
- **Formatting**: Check for consistency in formatting, particularly in the references section and the use of headings and subheadings.
- **Data presentation**: Remove the percentage signs if individual counting is being referred to or ensure the percentages are correct. For instance, the statement "Publications as review papers constitute 61% and 1% as conference papers" needs clarification or correction.
- **Language**: Correct typographical errors in the conclusion, such as changing "It study shows" to "This study shows."

**Recommendation:**

 The manuscript has potential and provides valuable insights into the research trends on functional ingredients in biscuits. However, it requires significant revisions to improve the depth of analysis, clarity of presentation, and overall impact. I recommend a **major revision** before reconsideration for publication.

Are the rationale for, and objectives of, the Systematic Review clearly stated?

Partly

Is the statistical analysis and its interpretation appropriate?

Yes

If this is a Living Systematic Review, is the ‘living’ method appropriate and is the search schedule clearly defined and justified? (‘Living Systematic Review’ or a variation of this term should be included in the title.)

Not applicable

Are sufficient details of the methods and analysis provided to allow replication by others?

Partly

Are the conclusions drawn adequately supported by the results presented in the review?

Partly

Reviewer Expertise:

food ingredients; food technology; food product development; food science and nutrition; functional foods; systematic literature reviews; data analysis; impact of food ingredients on health

I confirm that I have read this submission and believe that I have an appropriate level of expertise to confirm that it is of an acceptable scientific standard, however I have significant reservations, as outlined above.

# Response

**Published:** 2024-09-16

**Authors:** Varalakshmi Chandra Sekaran

**Affiliations:** Melaka Manipal Medical College, India

The literature review is updated as per the comments.

 The methodology is improved and justification is added for the selected method.

 The result section is updated as per the comment.

 The network analysis section is improved to incorporate changes.

 The revision is made to the discussion section.

 All the comments are looked into and necessary changes are made in the manuscript.
